# Mechanism of miR-107/HMOX1 axis in hepatic sinusoidal endothelial cells stimulated by ischemia-reperfusion injury

**DOI:** 10.1186/s41065-025-00495-4

**Published:** 2025-07-16

**Authors:** Zhao Wang, Jiangyang Sun, Yu Wang, Yichuan Zhang, Laian Ge

**Affiliations:** 1 College of Clinical Medicine, Jiangxi University of Chinese Medicine, Nanchang, 330006 China; 2 Department of Hepatobiliary Surgery, The Central Hospital of Wuhan, Tongji Medical College, Huazhong University of Science and Technology, Wuhan, 430014 China; 3 Minimally Invasive Endoscopy Center, Digestive Disease Center, The Affiliated Hospital of Panzhihua University, Panzhihua, 617000 China; 4 Department of Gastroenterology One, The Affiliated Hospital of Jiangxi University of Chinese Medicine, No. 445, Bayi Avenue, Donghu District, 330006 Nanchang, China

**Keywords:** miR-107, HMOX1, Hepatic IRI, Inflammation, Oxidation

## Abstract

**Background:**

Liver ischemia-reperfusion injury (IRI) is a common complication of diseases such as liver transplantation, hepatic resection, and hemorrhagic shock. This study aimed to elucidate the molecular mechanism of miR-107 affecting hepatic ischemia-reperfusion injury (IRI).

**Methods:**

The expression changes of miR-107 during hepatic IRI were quantified using quantitative real-time PCR. Subsequently, in vitro cellular experiments were carried out to verify the role of miR-107 on hypoxia/reoxygenation (HR) through CCK-8, flow cytometer, and commercial kits. In terms of mechanism, it was determined that miR-107 had a regulatory relationship with target genes through luciferase reporter assay.

**Results:**

In mouse liver IRI, miR-107 expression was increased while HMOX1 expression was decreased in liver tissues. In vitro cellular experiments, miR-107 inhibitors favored the alleviation of proliferation, apoptosis, inflammation, and oxidative stress in HR-damaged liver sinusoidal endothelial cells. In the molecular mechanism study, we determined that miR-107 could bind to HMOX1 and inhibit the HMOX1 expression. Low HMOX1 expression could eliminate the protective effect of miR-107 inhibitors.

**Conclusion:**

MiR-107 expression was elevated during hepatic IRI and exacerbates hepatic injury by targeting HMOX1 inhibition.

## Introduction

The increasing use of liver transplantation as a treatment for end-stage liver disease has been accompanied by attention to ischemia-reperfusion injury (IRI) that may be induced by liver transplantation [1]. Hepatic IRI may lead to postoperative hepatic dysfunction, affecting the prognostic outcome of transplanted liver grafts, exacerbating hepatic injury, and even triggering hepatic failure or multi-organ failure, which may threaten the patient’s life [2, 3]. Mitigating IRI in liver transplantation is important for postoperative recovery as well as the long-term prognosis of liver transplant patients. The mechanism of IRI is very complex, and it is a process that involves a variety of cells such as endothelial cells, immune cells, and parenchymal cells, as well as a variety of mechanisms such as endothelial damage, oxidative stress, inflammatory response, and apoptosis [4, 5]. The exact regulatory processes involved in the pathogenesis of hepatic IRI have not yet been elucidated; therefore, clarification of the factors associated with the pathogenesis of hepatic IRI is relevant to the mitigation of liver injury.

MicroRNAs are short non-coding RNA molecules, typically 21–25 nucleotides (nt) in length. They silence target genes either by inhibiting mRNA translation or by binding to the 3’-untranslated region (UTR) of mRNA, leading to mRNA degradation. MicroRNAs play a crucial role in various diseases, including cancer [6]. The expression of miR-34a-3p was significantly downregulated in oral squamous cell carcinoma (OSCC) tissues compared to normal tissues [7]. In addition, several miRNAs have been found to exert protective effects on ischemia/reperfusion injury of organs. For example, miR-21 is frequently upregulated in various types of stroke, indicating its significant role in regulating cell proliferation and apoptosis [8]. miR-1303a-3p exerts protective effects against brain injury in a cerebral ischemia-reperfusion rat model [9]. Inhibition of miR-186-5p expression prevented the–apoptotic percentage of hepatic cells, thereby reducing ischemic injury by hindering Yin Yang 1 [10]. MiR-107 is an important microRNA that plays an important role in liver-related diseases and tumors. Inhibition of miR-107-3p suppresses hg-induced proliferation and fibrosis, thereby contributing to the progression of diabetic nephropathy [11]. In addition, it has been reported that miR-107 induces apoptosis by participating in the I/R process. In animals with middle cerebral artery occlusion, the quantification of miR-107 is ascended and aggravates neurotoxicity [12]. The upregulation of miR-107 facilitates the apoptosis of models of cerebral IRI, reflecting that miR-107 may be a regulator of IRI. Given the role of miR-107 in IRI, we speculate that miR-107 may play an important role in hepatic IRI. Specific mRNAs are important for the function of miRNAs. Heme oxygenase-1 (HMOX1) is involved in ischemia-reperfusion injury in a variety of organs, in which it has a protective effect against liver injury and hepatocyte apoptosis [13, 14]. Database analysis reveals that HMOX1 was a downstream target gene of miR-107. Based on this evidence, we hypothesized that the miR-107/HMOX1 axis may be involved in the pathogenesis of liver IRI.

In the present study, we simulated the hepatic IRI animal models and liver sinusoidal endothelial cell (LSEC) models. The concentration of miR-107 was accurately determined in both models. The putative mechanism was raised in the LSEC models.

## Materials and methods

### Liver IRI animal models

This experimental scheme is authorized by the Animal Care and Use Committee of The Affiliated Hospital of Panzhihua University. Experiments were performed using 6–8-week-old C57BL/6J male mice (20–25 g, Cavens, Changzhou, China). A mouse warm liver IRI model was established following previous studies. Briefly, mice were anesthetized with sodium pentobarbital, and the blood supply to the left and middle hepatic lobes was blocked, resulting in 70% hepatic IRI. Experimental mice were subjected to ischemia for 1 h and reperfusion for 6 h. To maintain body temperature, mice were placed on a thermal blanket. Each mouse was housed and cared for individually after surgery. At the end of reperfusion, the injured liver lobes were removed at 3, 5, and 7 days, respectively. Liver tissue of each group 1/4 was ground by adding liquid nitrogen and added to Trizol liquid to extract RNA for backup; the remaining samples were used for histological studies. After obtaining samples, mice were euthanized by cervical dislocation. All these protocols followed principles of Animal Research: Reporting of In Vivo Experiments.

### HE staining, immunohistochemistry, and Immunofluorescence

The liver tissue was fixed using formalin and treated with xylene and alcohol. Hematoxylin and eosin dye were used to treat the tissues sequentially. After dehydration and transparency treatment, the samples were observed using a microscope.

The samples were completely covered with 5% goat serum and incubated in a 37 °C wet box for 30 min. The Lymphatic Vessel Endothelial Hyaluronan Receptor-1 (LYVE-1) antibodies (E3L3V Rabbit mAb) and proliferating cell nuclear antigen (PCNA) antibodies (PC10 Mouse mAb or XP Rabbit mAb D3H8P) were diluted separately with PBS and apply the diluted antibodies to the specimen to be examined. The diluted secondary antibody was added. The slide flat was placed in a wet box and held at 37 °C for 30 min. A drop of DAPI was added and covered with a coverslip. The results were visualized under a fluorescence microscope (Leica TCS SP8, Germany). All antibodies were from Cell Signaling Technology (USA).

### LSEC models of hypoxia/reoxygenation (HR)

The LSECs and Prigrow medium were purchased from Applied Biological Materials (Vancouver, BC, Canada). Oligonucleotides, including miR-107 controls (miR-NC: An artificially synthesized oligonucleotide with no complementarity to the target miR-107 sequence was used as a negative control), inhibitors, si-controls (si-NC: An artificially synthesized oligonucleotide sequence with no sequence homology and not targeting any specific mRNA was used as a negative control), and si-HMOX1 (all from ThermoFisher, America), were transfected into LSECs using Lipofectamine 2000 (ThermoFisher) 24 h before constructing cell models. Throughout the experiment, the cell culture medium was used with less than 5% serum and a temperature of 37 ℃. When the cell confluence reached 70%, the cell HR model was established. This was done as follows: cells were hypoxia-treated in a gas mixture containing 95% N_2_ and 5% CO_2_ for 6 h and then reoxygenated for 24 h using a gas mixture of 95% air and 5% CO_2_.

### qRT-PCR experiment

The Trizol method was used to extract total RNA from cells and liver-injured tissues. After the concentration was determined, the RNA concentration was diluted to 500 ng/microliter. The reverse transcription products were amplified using miRNA-specific forward primers and universal reverse primers, which were designed based on the criteria outlined in Primer Premier 5 (Premier Biosoft). A reverse transcription kit and qPCR kit (all from Transgen, Beijing, China) were adopted. Genomic DNA was removed before cDNA synthesis according to the manufacturer’s instructions. The relative expression of miR-107 and HMOX1 and the reference U6 and GADPH. The primer sequences were as follows: miR-107: F: 5’-GGAGCAGCATTGTACAGG-3’, R: 5’-CAGTGCGTGTCGTGGA-3’. Hmox1 F 5’-GGCCCTGGAAGAGGAGATA; R 5’-GCTGGATGTGCTTTTGGTG. U6: F: 5’-GCTTCGGCAGCACATATACTAAAAT-3’ and R: 5’-CGCTTCACGAATTTGCGTGTCAT-3’. GADPH: F 5’-TGAAGGTCGGAGTCAACGGATTTGGT; R 5’-CATGTGGGCCATGAGGTCCACCAC. Using a 20 µl reaction system as an example: the reaction mixture includes the template (amount adjusted as needed), 10 µM forward primer (0.4 µl, final concentration 0.2 µM), 10 µM reverse primer (0.4 µl, final concentration 0.2 µM), 2× PerfectStart^®^ Green qPCR SuperMix (10 µl, final concentration 1×), optional Universal Passive Reference Dye (50×, 0.4 µl, final concentration 1×), and nuclease-free water to adjust the total volume to 20 µl. The qPCR protocol consists of a pre-denaturation step at 94℃ for 30 s, followed by 40 cycles of amplification. Each cycle involves denaturation at 94℃ for 5 s, annealing at 50–60℃ for 15 s, and extension at 72℃ for 10 s. qPCR results were calculated using the 2^−∆∆CT^ formula.

### Proliferation and apoptosis

The LSECs of each group after different treatments were inoculated in 96-well plates (5 × 10^3^ cells/well), and 10 µL CCK-8 reagent was added to each well and incubated in the culture chamber for 2 h. The absorbance value of each well (A = 450 nm) was detected by the microplate reader.

The cells of each group were inoculated into 6-well plates, washed with pre-cooled PBS, resuspended with 500 µL of binding buffer, and then added with 5 µL of Annexin V-FITC and 5 µL of propidium iodide (PI), and then incubated for 15 min at room temperature under room temperature and light, and the apoptosis rate was detected by flow cytometer.

### Targeted relationship

The putative relationship between HMOX1 and miR-107 was researched on RNAhybrid. A dual luciferase reporter assay was used to verify the relationship between miR-107 and HMOX1. miR-107 control or inhibitor and psi-CHECK2-HMOX1 were co-transfected. After 36 h of transfection, cells were removed and washed with PBS. After 48 h of transfection, the cells were removed, the medium was discarded, and the cells were washed again with PBS. The fluorescence activity of each group was measured using a fluorescence intensity quantification kit (Saint-Bio, Shanghai, China).

### Biochemical detection

Tumor necrosis factor (TNF)-а, interleukin-6 (IL-6), and interleukin-1β (IL-1β) were detected by enzyme-linked immunosorbent assay (ELISA), and the kits were obtained from Jingmei (Beijing, China). Malondialdehyde (MDA), superoxide dismutase (SOD), and catalase (CAT) activities were determined by test kits provided by Langkang (Shanghai, China).

### Statistical analysis

GraphPad software was used for data processing and plotting. Independent sample t-test was used for comparison between the two groups, and one-way analysis of variance was used for comparison between multiple groups. Statistical differences were represented as *P* < 0.05.

## Results

### Successful construction of animal models

**Fig. 1:**
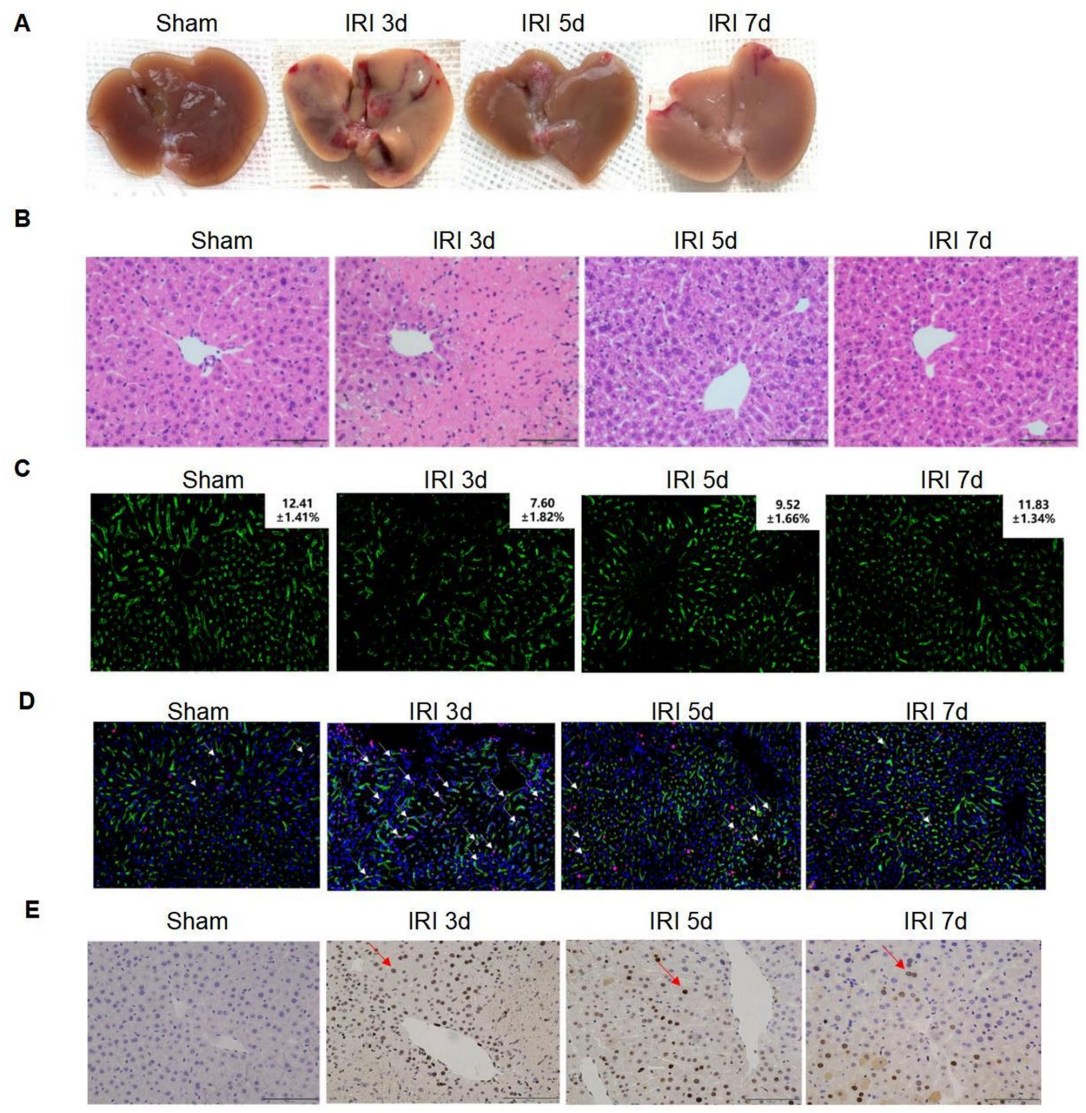
The experimental results graph of hepatic IRI vividly illustrates the pathological progression of liver damage by comparing key metrics at distinct time points (sham operation, IRI 3 days, 5 days, 7 days). **(A)** The general appearance of the liver (visual observation): As time progresses, the liver exhibits increasing signs of congestion, swelling, and localized necrosis (e.g., dark purple regions), which suggests that ischemia-reperfusion injury (IRI) induces pronounced morphological alterations in the liver. The severity of damage appears to peak between 3 and 5 days. (**B**) HE staining (histopathological morphology, light microscopy observation) confirmed that ischemia-reperfusion injury (IRI) induced significant pathological damage to liver cells. (**C**) The results of tissue immunofluorescence LYVE-1 labeling of LSECs. (**D**) Tissue immunofluorescence labeling with LYVE-1 and PCNA. (**E**) Immunohistochemistry results using PCNA

By observation of the liver, the extent of liver damage was obvious after 3 days of IRI in mice, and the liver loss recovered on day 5, with the best recovery after day 7 (Fig. 1A). HE staining results showed that the pathological damage was the most severe on day 3, and the damage was reduced from day 5 to day 7 (Fig. 1B). The results of tissue immunofluorescence LYVE-1 labeling of LSECs were exhibited in Fig. 1C, indicating that LSECs showed a large amount of necrosis and severe destruction of the liver sinusoidal structure after 3 days of IRI, and the liver sinusoidal structure was gradually recovered on the fifth day, and the destruction was the lightest on the seventh day. Tissue immunofluorescence labeling with LYVE-1 and PCNA was adopted to reflect the proliferative stage of LSECs, which showed significant proliferation on day 3 after IRI (Fig. 1D). Immunohistochemistry using PCNA also showed that hepatocytes proliferated significantly only on the third day after IRI (Fig. 1E).

### Change in expression levels

**Fig. 2:**
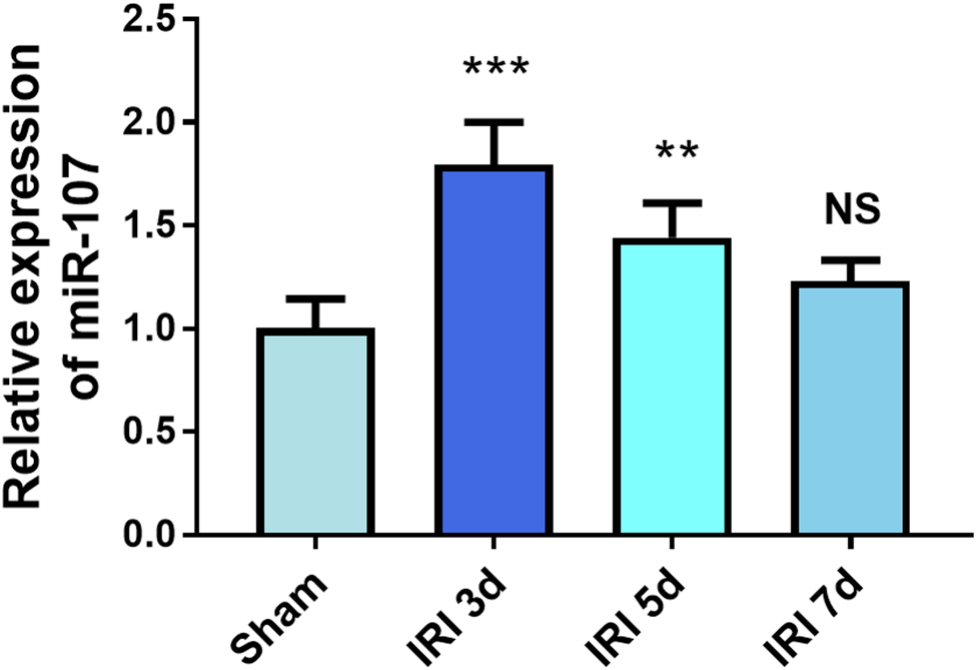
The IRI treatment induced the upregulation of miR-107 expression. **P < 0.01, ***P < 0.001, compared to sham group

The content of miR-107 was increased in the IRI 3d group and the IRI 5d group, suggesting that the increased miR-107 might be led by liver IRI damage (*P* < 0.01, Fig. 2). The expression of miR-107 represented an elevated trend during the period from day 3 to day 7 after liver IRI (Fig. 2).

### Anti-protective impacts of miR-107

**Fig. 3:**
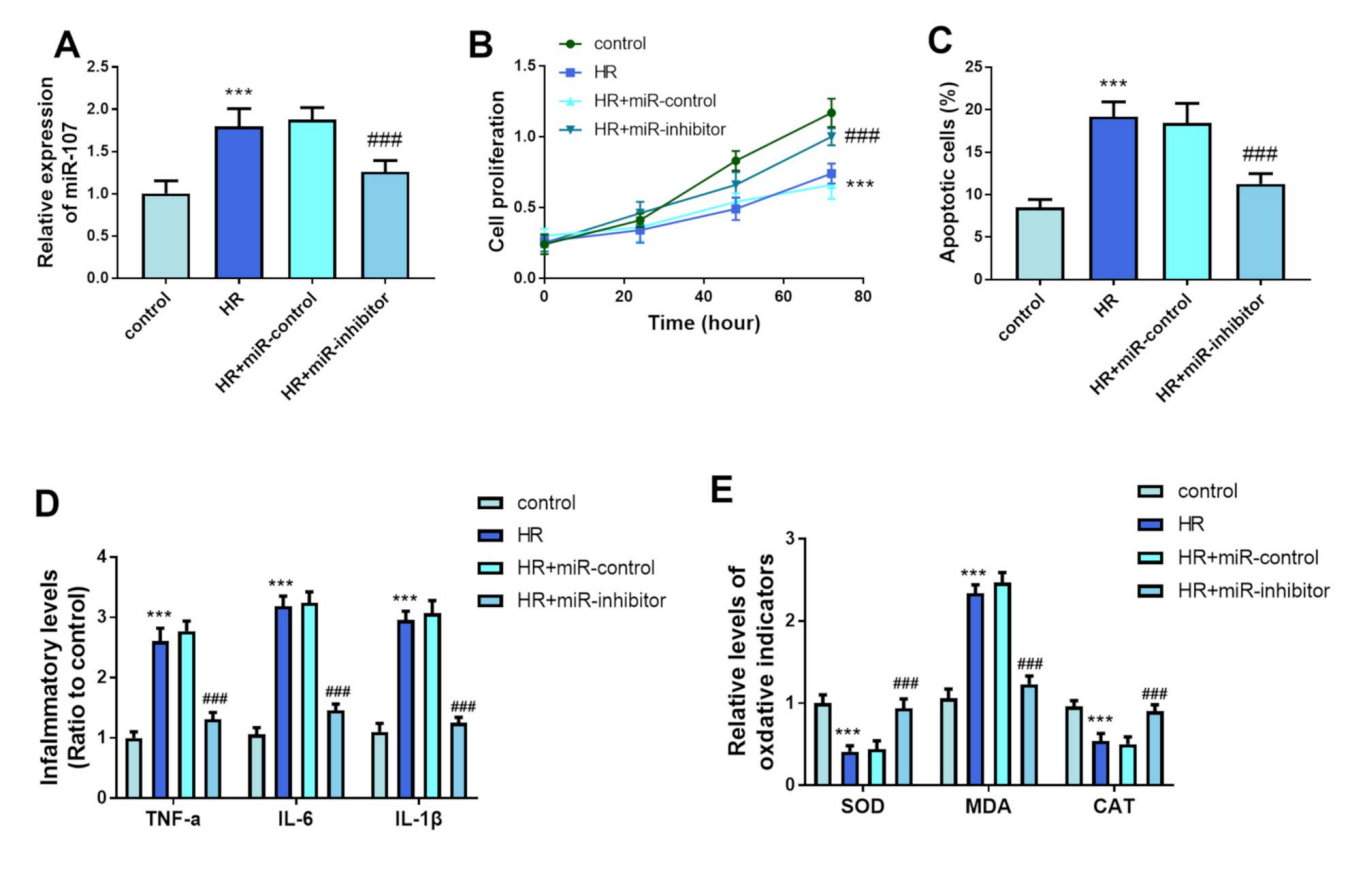
Significance of miR-107 on LSECs. (**A**) H/R treatment induced the significant upregulation of miR-107, and its inhibitor could effectively suppress this upregulation. **(B)** Proliferative ability was recovered after silencing miR-107. (**C**) Knockdown of miR-107 reversed the abnormal apoptosis in the HR group. (**D-E**) Protective roles of silenced miR-107 on inflammation and oxidation. ***P < 0.001, compared to control group; ###*P* < 0.001, compared to HR group

An increase in miR-107 concentration was observed in the HR group and it was lessened in the HR + miR-inhibitor group (*P* < 0.001, Fig. 3A). The proliferative rate of the HR group was suppressed, while the knockdown of miR-107 exerted a reversed function, which moderated the impacts of HR on cell proliferation (*P* < 0.001, Fig. 3B). The increase in apoptotic percentage in the HR group was prevented by the declined miR-107 concentration (*P* < 0.001, Fig. 3C). The inflammatory response was increased in the HR group and reversed in the HR + miR-inhibitor group (*P* < 0.001, Fig. 3D). The reduced SOD increased MDA and declined CAT reflected the oxidative stress of LSECs induced by HR (*P* < 0.001, Fig. 3E). The silenced miR-107 inhibited the oxidation (*P* < 0.001, Fig. 3E).

### HMOX1 and miR-107 interconnection

**Fig. 4:**
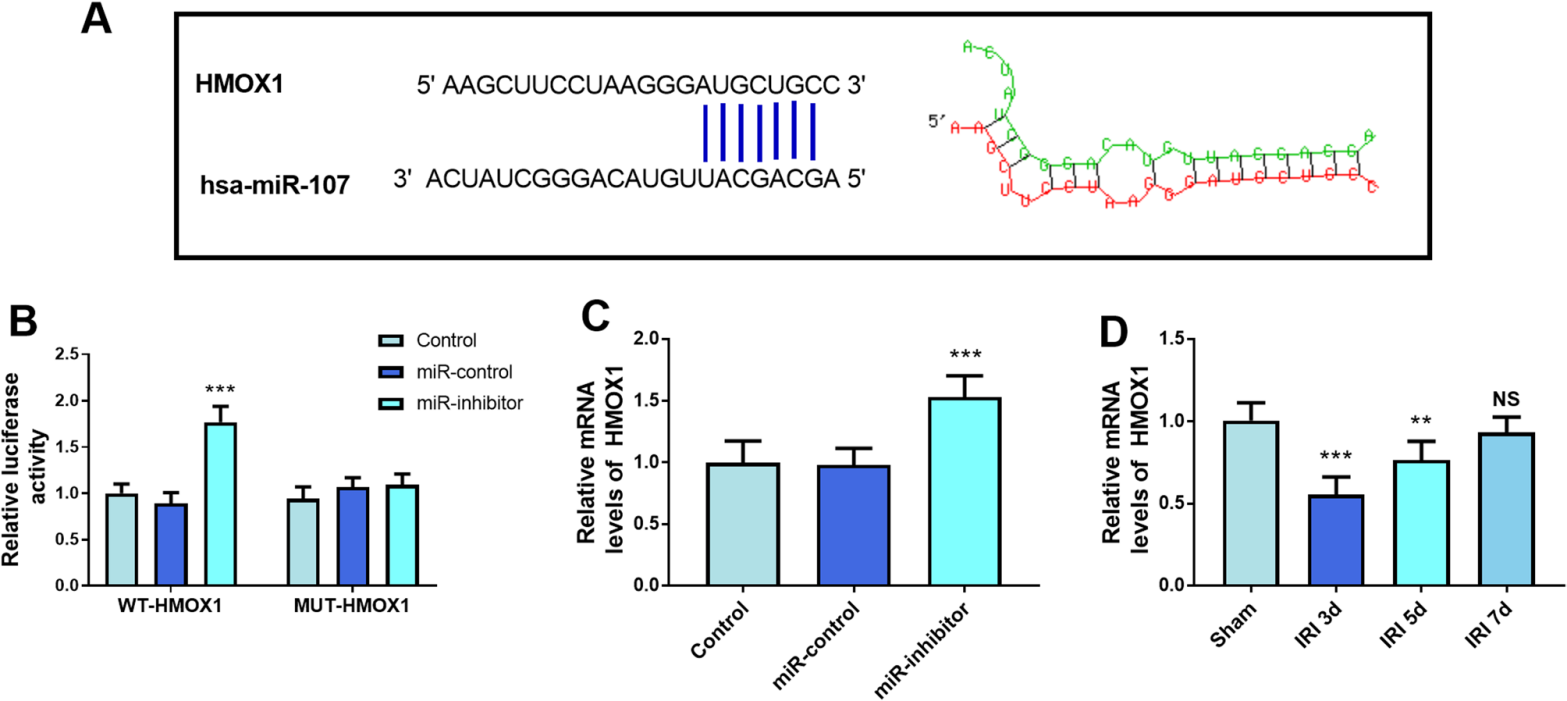
miR-107 regulated HMOX1 expression. (**A**) The target binding sites of miR-107 and HMOX1. (**B**) The dual-luciferase reporter assay was employed to validate the direct targeting relationship between miR-107 and HMOX1. (**C**) miR-107 negatively regulated the expression level of HMOX1. (**D**) The expression levels of HMOX1 induced by IRI. ***P < 0.001, compared to the control group or sham group

The putative bases between HMOX1 and miR-107 were documented in Fig. 4A. The reduced miR-107 concentration of the WT-HMOX1 group promoted the luciferase activity, pinpointing that miR-107 might target HMOX1 (*P* < 0.001, Fig. 4B). Additionally, the mRNA levels of HMOX1 were enhanced by the inhibition of HMOX1 (*P* < 0.001, Fig. 4C). The amount of HMOX1 was reduced in the IRI 3d and IRI 5d, compared to the sham group (*P* < 0.01, Fig. 4D). After hepatic IRI, the HMOX1 gradually increased from low expression to near-normal levels (Fig. 4D).

### Axis HMOX1/miR-107 on LSEC models

**Fig. 5:**
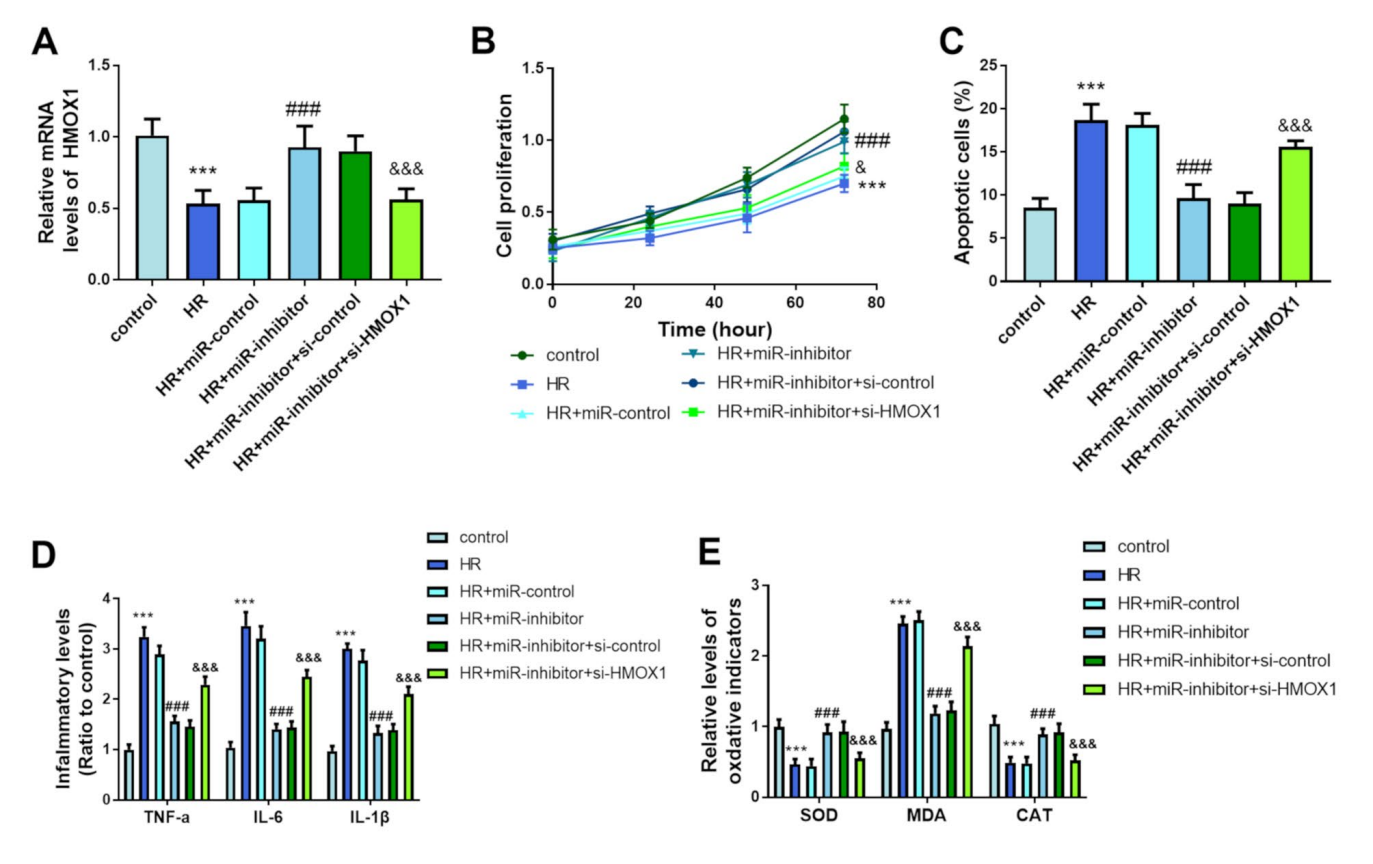
The miR-107/HMOX1 axis plays a regulatory role in LSECs. (**A**) Regulation of HMOX1 levels under different treatments. (**B-E**) miR-107 modulates proliferation, apoptosis, inflammation, and oxidation in LSECs by directly targeting HMOX1. ***P < 0.001, compared to control group; ###*P* < 0.001, compared to HR group; &*P* < 0.05, &&&*P* < 0.001, compared to HR + miR-inhibitor group

The cotransfection of miR-107 inhibitor and si-HMOX1 to the LSECs reversed the increased mRNA levels of HMOX1 (*P* < 0.001, Fig. 5A). The decreased HMOX1 reversed the impacts of miR-107 inhibitor on the proliferation and apoptosis of LSECs (*P* < 0.05, Fig. 5B-C). The knockdown of HMOX1 alternated the inflammatory situation and oxidative cytokines of the HR + miR-inhibitor group, indicating that HMOX1 is a downstream regulator in the regulation of miR-107 (*P* < 0.001, Fig. 5D-E).

## Discussion

Ischemia-reperfusion injury is very common in clinical practice, such as liver transplantation, liver resection, and liver trauma [15]. During liver surgery, to minimize intraoperative bleeding, blood flow to the liver from the hepatic hilum is often blocked, and the blood supply is restored after the surgery [16]. When the blood supply is restored after surgery, hepatic I/R occurs, which leads to hepatic I/R injury. As endogenous regulators of gene expression, many miRNAs are involved in the internal regulation of liver IRI [17, 18]. The alternation of miR-181a-5p and YY1 expression in the mice IRI models and HR hepatocyte models indicates their regulation in IRI [18]. MiR-450b-5p aggravates the liver IRI and function by promoting inflammatory cytokines and inhibiting the levels of CRYAB [19]. Taken together, specific miRNAs took part in hepatic IRI and are of great importance in revealing the IRI mechanism.

In the present observation, the mouse IRI models were established and the pathological examination documented the IRI damage reached its maximum severity on day 3 and gradually recovered thereafter. The expression of miR-107 represented a similar changed trend with the severity of IRI, which showed the highest levels after 3 days of IRI treatment and then gradually decreased. The upregulation of miR-107 is involved in the recovery of cerebral IRI in rats, indicating that miR-107 may regulate IRI [20]. In the pigs that received lung autotransplantation, the generation of miR-107 was promoted due to lung IR, pinpointing that miR-107 is a common molecular in IRI [21].

LSECs have the highest percentage of nonparenchymal cells and also play a key role in liver repair and regeneration [22]. The HR treatment induced the LSEC-damaged models and elevated miR-107 levels. The repressed miR-107 reversed the destructive roles of HR on proliferation, apoptosis, inflammation, and oxidative of LSECs, underscoring the protective impacts of miR-107 knockdown. The occurrence of IRI caused the activity of inflammatory cytokines. In sepsis-induced lung injury, the upregulation of miR-107 facilitates the presence of proinflammatory cytokines, which pinpoints the implication of miR-107 on inflammation [23]. The increased miR-107 aggravates the tubular cell injury by promoting the production of TNF-а and inhibiting the target DUSP7 [24]. In this article, we demonstrated that HR caused abnormal expression of inflammatory factors, and the silencing of miR-107 reversed this phenomenon, indicating that miR-107 was closely related to the inflammatory response of hepatic IRI. In hepatic IRI, the hypoxic environment can lead to inactivation or depletion of endogenous antioxidants or enzymes, along with the production of the harmful substance MDA [25]. miR-107 exhibits a facilitating effect on the oxidative stress response [26]. The silenced miR-107 protected LSECs against damage from oxidative stress, manifesting that miR-107 might regulate hepatic IRI by the way of manipulating oxidative factors.

In this observation, the targeted correlation between miR-107 and HMOX1 was supported by the luciferase reporter assay. The expression of HMOX1 declined in liver IRI and gradually elevated during the recovery of liver damage. HMOX1, also known as heat shock protein-32, is a microsomal enzyme that plays an important role in oxidative stress, inflammation, and other processes [27]. In hepatocellular carcinoma, the levels of miR-107 are regulated by HMOX1, and their axis participates in the progression of malignancy [28]. Previous reports have indicated that HMOX1 is involved in the regulation of hepatic IRI by a variety of mechanisms, including antioxidant regulation and inflammatory properties [14, 29]. The silenced HMOX1 represented a disadvantageous impact on the proliferation, apoptosis, inflammation, and oxidation of LSECs, and it mediated the regulation of miR-107 in these respects. Collectively, the miR-107/HMOX1 axis might participate in the regulation of liver IRI. HMOX1 may exert protective effects via multiple downstream pathways, including regulation of oxidative stress (Nrf2/ARE signaling, ROS scavenging) [30, 31], modulation of inflammatory responses (NF-κB inhibition, macrophage M2 polarization, NLRP3 inflammasome suppression) [32–34], and control of apoptosis (mitochondrial-mediated pathway, PI3K/Akt signaling activation, JNK cascade inhibition) [35–37]. miR-107 suppresses HMOX1 expression by targeting its 3’UTR, thereby attenuating the functions of HMOX1-related antioxidant (Nrf2/ARE signaling, ROS scavenging), anti-inflammatory (NF-κB inhibition, macrophage M2 polarization), and anti-apoptotic (PI3K/Akt signaling activation) pathways. Consequently, this exacerbates cellular damage in liver IRI. Nevertheless, further investigation is required to elucidate the interactions among these mechanisms in liver IRI, refine the regulatory network of the miR-107/HMOX1 axis, and establish a robust theoretical foundation for clinical translation.

Clinically, the treatment plan for patients with liver IRI should consider the expression levels of miR-107 and HMOX1. miR-107 was upregulated during liver IRI and exacerbated liver damage by targeting and inhibiting HMOX1. Therefore, therapeutic strategies targeting miR-107 or HMOX1 may benefit patients with liver IRI, such as using miR-107 inhibitors to suppress its expression or enhancing HMOX1 expression via alternative approaches. These interventions could alleviate pathological processes including oxidative stress, inflammation, cell proliferation, and apoptosis, thereby improving patient prognosis. While these findings may not immediately alter existing therapies, they provide novel targets for developing precise intervention strategies.

Furthermore, this study did not perform rescue experiments involving miR-107 inhibition or HMOX1 overexpression in animal models to further validate the functional causal relationship between miR-107 and HMOX1 in liver IRI. This represented one of the limitations of this study. In future research, additional in vivo functional intervention experiments will be carried out to confirm the causal relationship of the miR-107/HMOX1 axis in liver IRI through strategies such as miR-107 inhibition, HMOX1 overexpression, and rescue experiments. Furthermore, the in vivo mechanisms of HMOX1’s downstream signaling pathways will be elucidated, and the applicability of the animal model for clinical translation will be optimized. Another limitation of this study is that it only focused on liver sinusoidal endothelial cells (LSECs). The collaborative interactions among hepatocytes, Kupffer cells, and neutrophils were overlooked, making it challenging to fully capture the true hepatic microenvironment, including the role of the miR-107/HMOX1 axis.

Future studies could construct multi-cell models by co-culturing hepatocytes with LSECs or immune cells with parenchymal cells, thereby simulating the liver sinusoidal structure and immune cell activation. Such models would enable the analysis of intercellular signaling interactions, the heterogeneity and spatial co-expression patterns of miR-107/HMOX1 across different cell types, as well as the expression differences of this axis in multiple cell types and its correlation with inflammatory infiltration. These analyses would systematically uncover the collaborative regulatory network between cells and enhance the translational relevance of the research. Moreover, traditional Chinese medicine (TCM) offers promising benefits, such as immune regulation, neuropsychological support, liver protection, and enhancing the quality of life [38]. By embracing the holistic approach and personalized care that TCM provides, we can improve patient outcomes, reduce the burden on healthcare providers, and tackle long-term health challenges like metabolic dysfunction. Looking ahead, it would be valuable to explore emerging integrative approaches in liver disease management, which could further enhance the recovery prospects for liver transplant patients. Future research also can further expand into the field of computational biology. Generative Adversarial Networks (GANs) provide a novel research paradigm for analyzing complex transcriptional regulatory mechanisms [39]. By generating synthetic gene expression data, GANs can effectively overcome the problem of limited clinical sample size, thereby deepening the analysis of the miR-107/HMOX1 dynamic regulatory network. At the same time, with its simulation capabilities, it can reconstruct the signal transmission process in the multi-cellular microenvironment and reveal the synergistic mechanism between liver sinusoidal endothelial cells (LSECs) and surrounding cells in ischemia-reperfusion injury. Moreover, GANs can also be used to predict off-target effects that may arise from targeted interventions, thereby accelerating the optimization process of treatment strategies. The combination of experimental methods and computational models is expected to promote the research on the mechanism of liver ischemia-reperfusion injury to the level of systems biology.

This study investigates the linear regulatory relationship between miR-107 and the protein-coding gene HMOX1. However, the biological functions of miRNAs were frequently integrated into more complex non-coding RNA networks. Recent studies have highlighted that lncRNAs can modulate gene networks during stress responses, such as ischemia-reperfusion injury, either by competing with miRNAs (the ceRNA mechanism) or by regulating transcription factors [40–43]. Future research could explore whether specific lncRNAs regulate miR-107 in liver IRI, as well as how the gene-lncRNA-miRNA ternary network collaboratively influences the antioxidant and anti-inflammatory pathways downstream of HMOX1. Adopting this network-based perspective will enhance our understanding of the systemic-level function of miR-107 within the liver microenvironment. The current discovery of miR-107’s regulatory role in liver IRI can serve as an “anchor point” for interdisciplinary technological applications. Specifically, AI could be employed to elucidate its functions in other liver diseases, such as liver fibrosis and hepatocellular carcinoma. Multi-omics integration may further refine the underlying regulatory mechanisms, while bioinformatics approaches could facilitate the translation of these findings into clinical applications [44]. Looking ahead, leveraging advanced tools like AI and multi-omics technologies will expedite the transition toward precision diagnosis and treatment, potentially revolutionizing the management of liver diseases. These efforts aim to provide clearer molecular targets and a stronger theoretical foundation for the treatment of liver IRI.

In summary, elevated miR-107 and reduced HMOX1 were observed in the liver tissues of IRI mice models. MiR-107 exacerbates hepatic IRI expression from oxidation, inflammation, proliferation, and apoptosis by targeting HMOX1. This study has for the first time demonstrated the pivotal role of the miR-107/HMOX1 axis in hepatic IRI. It extended the understanding of miRNA and HMOX1 functions, offering novel targets for elucidating the mechanisms underlying liver IRI and developing therapeutic strategies. The novelty of this work lies in bridging the research on miR-107 from tumor biology and neurology to hepatic pathology, with a specific focus on LSECs, an underexplored cell population. This approach not only enriched the mechanistic insights into liver IRI but also may establish a foundation for multi-dimensional investigations into its pathophysiology.

## Data Availability

The datasets used and/or analyzed during the current study are available from the corresponding author upon reasonable request.
